# Slight pH Fluctuations in the Gold Nanoparticle Synthesis Process Influence the Performance of the Citrate Reduction Method

**DOI:** 10.3390/s18072246

**Published:** 2018-07-12

**Authors:** Braulio Contreras-Trigo, Víctor Díaz-García, Enrique Guzmán-Gutierrez, Ignacio Sanhueza, Pablo Coelho, Sebastián E. Godoy, Sergio Torres, Patricio Oyarzún

**Affiliations:** 1 Facultad de Ingeniería y Tecnología, Universidad San Sebastián, Lientur 1457, Concepción 4080871, Chile; bcontrerast@docente.uss.cl (B.C.-T.); victor.diazg@uss.cl (V.D.-G.); 2 Facultad de Ciencias de la Salud, Universidad San Sebastián, Lientur 1457, Concepción 4080871, Chile; enrique.guzman@uss.cl; 3 Departamento de Ingeniería Eléctrica, Facultad de Ingeniería, Universidad de Concepción, Concepción 4030000, Chile; ignacio.sanhueza.ruiz@gmail.com (I.S.); pcoelho@udec.cl (P.C.); segodoy@udec.cl (S.E.G.); sertorre@udec.cl (S.T.)

**Keywords:** gold nanoparticles, citrate reduction method, pH-effect, concentration

## Abstract

Gold nanoparticles (AuNPs) are currently under intense investigation for biomedical and biotechnology applications, thanks to their ease in preparation, stability, biocompatibility, multiple surface functionalities, and size-dependent optical properties. The most commonly used method for AuNP synthesis in aqueous solution is the reduction of tetrachloroauric acid (HAuCl_4_) with trisodium citrate. We have observed variations in the pH and in the concentration of the gold colloidal suspension synthesized under standard conditions, verifying a reduction in the reaction yield by around 46% from pH 5.3 (2.4 nM) to pH 4.7 (1.29 nM). Citrate-capped AuNPs were characterized by UV-visible spectroscopy, TEM, EDS, and zeta-potential measurements, revealing a linear correlation between pH and the concentration of the generated AuNPs. This result can be attributed to the adverse effect of protons both on citrate oxidation and on citrate adsorption onto the gold surface, which is required to form the stabilization layer. Overall, this study provides insight into the effect of the pH over the synthesis performance of the method, which would be of particular interest from the point of view of large-scale manufacturing processes.

## 1. Introduction

Gold nanoparticles (AuNPs) have emerged as a promising platform for a growing number of biomedical and biotechnology applications in the fields of sensing [1], molecular diagnostic [2], therapeutic [3], and imaging [4], owing to their stability, biocompatibility, remarkable physicochemical properties, and easy surface functionalization with a wide range of ligands [5,6]. A prominent optic feature of AuNPs arises from the collective oscillation of the conduction electrons in the presence of an incident light, the so-called surface plasmon resonance (SPR) [7]. This phenomenon causes a sharp and intense absorption band in the visible range, which can be readily tuned by varying the particle size, shape, and the surrounding physicochemical environment [8]. Thus, label-free colorimetric sensors based on AuNPs (nanobiosensors) have been widely proposed as a promising analytical for selective binding and detection of chemical and biological targets, including metal ions [9], antibiotics [10], mycotoxins [11], as well as a large number of microorganisms [12].

As concerns the preparation of the colloidal gold nanoparticles, various chemical routes, including the use of chemical reductants [13] and several photochemical methods based on UV irradiation [14], γ-irradiation [15], and laser irradiation [16], have been widely studied for different purposes of application. However, the classical “citrate reduction method” proposed by Turkevich in 1951 [17], and latter modified by Frens in 1973 [18], remains the most widely employed synthesis procedure, since AuNPs can be produced in a straightforward manner to obtain highly stable monodisperse particles with uniform spherical shape and narrow-size distributions ranging between 10–20 nm in diameter. This method is based on the aqueous-phase reduction of an Au^3+^ precursor (HAuCl_4_; gold salt) with sodium citrate near the boiling point of the reaction mixture, which produces a stable solution of metallic gold nanoparticles (colloidal gold) [19]. Recently, the use of citrate containing redox active ionic liquids has proved effective for simple synthesis of stable metal nanoparticles [20].

Citrate ions play a key role, since they act both as a reductant, converting gold ions (Au^3+^) into gold atoms (Au^0^), and as a protective agent that stabilizes the formed nanoparticles, preventing particle growth and aggregation via electrostatic repulsion (citrate capped AuNPs) [21]. Thus, at high citrate concentrations, smaller particles are covered and stabilized by this ion, while at low concentrations particle growth continues due to incomplete coverage, leading to the formation of AuNPs with larger particle size. A third role of citrate is as a mediator of the reaction mixture pH, through which it has a dramatic effect on the size, polydispersity, and morphology of the resulting AuNPs [22,23]. pH control on the citrate reduction method has been widely explored regarding its relationship with the size distribution of the AuNPs [24]. However, up until now the correlation of this parameter with the concentration of the resulting nanoparticles had not been described, which is of particular interest for citrate mediated synthesis of colloidal AuNPs to meet large scale manufacturing criteria [25].

**Figure 1:**
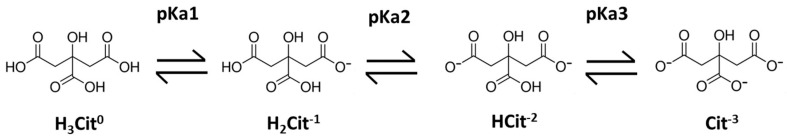
Chemical equilibria of citrate in aqueous solution.

The acid-base behavior of the AuNPs is provided by the citrate layer, which imparts a negative charge onto the colloidal particle surface. Citrate is a tricarboxylic acid (polyanion) with three pKa values (pKa1 = 3.06, pKa2 = 4.74, and pKa3 = 5.4) [26], which participate in the following chemical equilibria (Figure 1):

In this study, we investigated the relationship between typically occurring pH fluctuations of the colloidal suspension (between 4.5 and 5.3) and the concentration of the gold nanoparticles, providing valuable information regarding the synthesis performance of the method. AuNPs were characterized in terms of their concentration (optical density determinations), morphology, and surface charge, by carrying out transmission electron microscopy (TEM) examination, scanning electron microscopy with energy dispersive X-ray spectroscopy (EDS), and zeta potential analysis.

## 2. Materials and Methods

### 2.1. Preparation of Gold Nanoparticles (AuNPs)

Synthesis of AuNPs was carried out according to the procedures described in the citrate reduction method [17,18]. Briefly, 0.0394 g of tetrachloroauric acid (HAuCl_4_·3H_2_O) (Sigma Aldrich Chemical Company, Atlanta, GA, USA) was dissolved in 100 mL of nanopure water (18 MΩ of resistance) in a three-neck round flask (1 mM HAuCl_4_) connected to reflux condenser. The resulting solution was isovolumetrically heated to boiling point under stirring and refluxed. Then, 10 mL of a 38.8 mM trisodium citrate solution was preheated to 60 °C and quickly added to the boiling solution of HAuCl_4_ under vigorous stirring. After the solution turned from pale yellow to black and to deep red, it was refluxed for additional 30 min and subsequently cooled to room temperature without stirring for at least 2 h. The formed nanoparticle suspension was filtered through Millipore Nylon filter (0.45 µm) and preserved in the dark at 4 °C for subsequent pH determination and characterization of the AuNPs. 

### 2.2. AuNP Concentration

The concentration of gold synthesized under different pH conditions was determined by UV-Vis spectroscopy using an Epoch^TM^ Microplate Spectrophotometer (Bio-Tek Instruments, Winooski, VT, USA). 100 µL of each sample were transferred in duplicate into the microplate wells and the absorption spectra was recorded in the visible region (400 to 700 nm). The absorption maximum at the SPR band (520 nm) was employed to calculate the AuNP concentration according to the Beer-Lambert law, by using an extinction coefficient (ε) of 2.01 × 10^8^ M^−1^ cm^−1^ [27].

### 2.3. Electron Microscopy Analysis

The morphology and size of the AuNPs was determined by transmission electron microscopy with 4 Å resolution (TEM; JEOL-JEM 1200EX-II, Tokyo, Japan), using a Gatan CCD camera for image acquisition (model 782; Gatan, Inc., Pleasanton, CA, USA). The AuNPs samples were drop-cast on formvar/carbon-coated 200 mesh copper grids, which were subsequently retracted and allowed to dry in air at room temperature. Particle sizes and frequency histograms were obtained by measuring the diameter of 100 nanoparticles using the ImageJ software [28], while the percentage of spherical particles was determined through visual inspection of 100 nanoparticles. In addition, elemental analysis of the gold colloidal suspensions was carried out by energy-dispersive X-ray analysis (EDS system; Oxford Instruments, Oxford, UK) using scanning electron microscopy with a resolution of 133eV (SEM; JEOL JSM 6380LV, Tokyo, Japan). 

### 2.4. Surface Charge Characterization (pZ)

Zeta-potential measurements of AuNPs were measured on a zeta-potentiometer (Nano-ZS90, Malvern Instruments, Westboroug, MA, USA) at room temperature and scattering angle of 90° and 1 cm pathlength. A diluted suspension of AuNPs (100 µL diluted to 1 mL nanopure water) were employed for the analyses. The Malvern Zetasizer Software version 7.12 was employed to analyze the collected data.

## 3. Results and Discussion

Citrate-capped AuNPs were synthesized by the citrate reduction method and immediately characterized in terms of the pH of the solutions, as well as by UV-visible spectroscopy, size, morphology, elemental composition, and surface charge properties.

### 3.1. pH Effect on the Concentration

**Figure 2:**
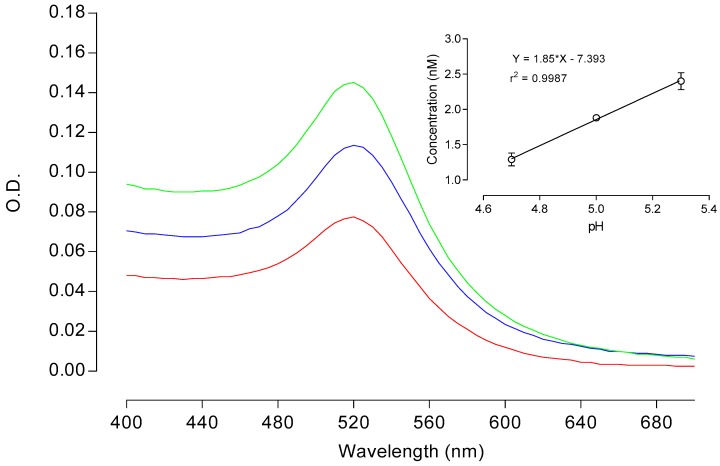
Absorption spectra of gold nanoparticles (AuNPs) obtained by the citrate reduction method at pH 4.7 (red line), 5.0 (blue line), and 5.3 (green line). The inset shows the linear correlation curve between pH and AuNP concentration is presented. Optical densities (O.Ds) correspond to the mean values calculated from independent determinations (*n* = 2).

Figure 2 presents the absorption spectra of the AuNPs solutions synthesized at variable pH values (4.7, 5.0, and 5.3), showing the typical curve with a characteristic maximum at 520 nm associated to the SPR band. However, differences in the absorbance values at this peak revealed that pH of the synthesis medium had a relevant effect on the resulting concentration of the nanoparticles. The AuNPs concentration varied in a directly proportional manner as a function of the pH (positive correlation), reaching pH 5.3 and about 1.8-fold the concentration (2.4 nM) than the one obtained at pH 4.7 (1.29 nM) (see Table 1). This correlation is highly linear within the monitored pH range (4.7–5.3), with a correlation coefficient (r^2^) of 0.9987.

### 3.2. AuNPs Characterization

**Figure 3:**
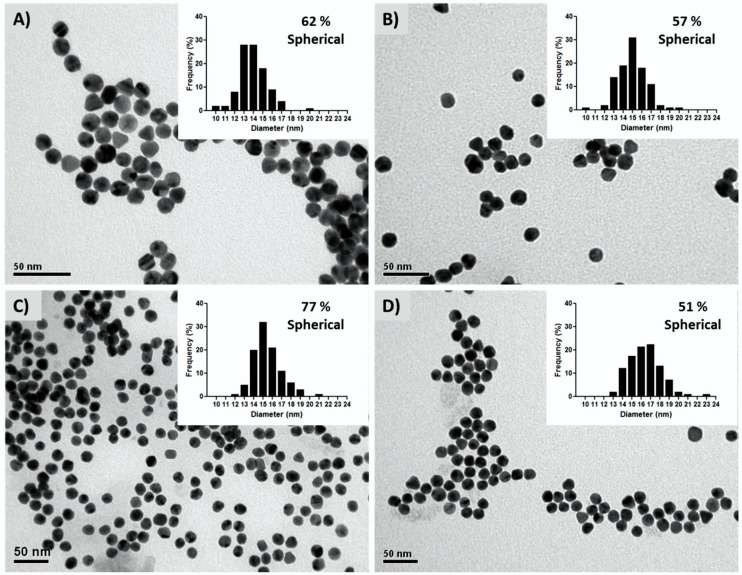
Transmission electron microphotographs of AuNPs at pH 4.7 (**A**), 5.0 (**B**), and 5.3 (**C**), and commercial AuNPs (pH 5.0) with an average diameter of 16.42 ± 1.76 nm (**D**). Histograms with the respective particle size distributions and the percentage of spherical nanoparticles are included as insets within each microphotography.

TEM analysis of the AuNPs samples proved the nanoparticles have comparable diameters between 13.9–15.5 nm for the tree monitored pH values, with a similar morphology distribution (Figure 3; Table 1). The percentage of spherical nanoparticles was slightly higher at pH 5.3 (77%) when compared to the percentage obtained with pH 5.0 (57%) and pH 4.7 (62%), which would favor shape homogeneity of the nanoparticles. By contrast, commercial AuNPs showed the least sphericity (51%). These observations are consistent with the optical behavior exhibited by the nanoparticle suspensions, which share identical wavelength associated to the SPR peak (UV-Vis spectrograms, Figure 2).

**Figure 4:**
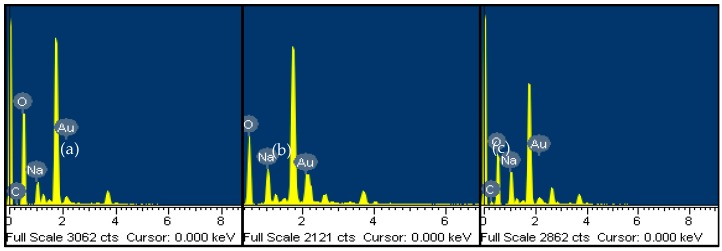
Energy dispersive X-ray spectrometry (EDS) spectra of gold colloidal suspensions at pH 4.7 (**a**), 5.0 (**b**), and 5.3 (**c**). Highest peaks (not labeled on the spectra) correspond to the silicon from the glass supports.

In addition, energy dispersive X-ray spectrometry (EDS) elemental analysis confirmed the presence of Au, C, O, and Na forming part of the citrate layer adsorbed on the AuNPs (Figure 4). Sodium ions play an important role in stabilizing high capping of the gold surface by carboxylate anions [29].

### 3.3. Surface Charge Analysis

Under standard experimental conditions employed in the present study the binding of citrate ions onto the surface of AuNPs occurs electrostatically through negative oxygen atoms of carboxylic groups (oxyanions) at the ends of the citrate molecule [29]. Thus, at pH 4.7 (close to pKa2) similar amounts of H_2_Cit^−1^ and HCit^−2^ implies a lesser availability of deprotonated carboxylic groups, while at pH 5.3 (close to pKa3) the dominant species HCit^−2^ and Cit^−3^ would determine a greater capability of citrate anions to coordinate to the metal surface. 

**Figure 5:**
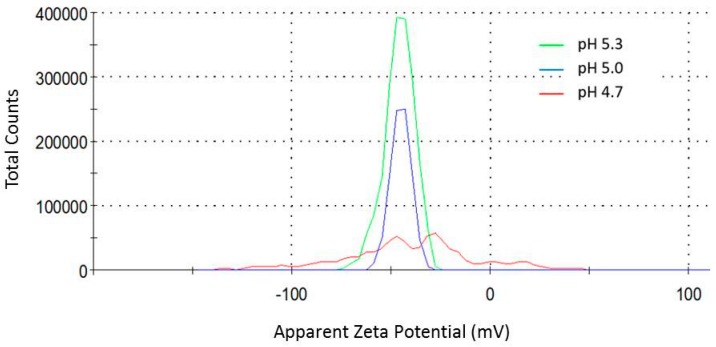
Zeta potential distribution (mV) of citrate-capped AuNPs synthesized at pH 4.7 (red), 5.0 (blue), and 5.3 (green).

The negative charge of citrate-stabilized AuNPs was confirmed through zeta potential analysis (pZ), revealing the influence of pH on the surface charge from the distribution of the pZ values (Figure 5). Thus, as the monitored pH fluctuated from 4.7 to 5.3, the shape of the curves reveals a progressive narrowing on the dispersion of the pZ values of the AuNPs, which accounts for a greater stabilization of the nanoparticles as a consequence of the presence of mostly deprotonated citrate anions.

The lowest dispersion values were determined at pH 5.0 and 5.3 (−44.9 ± 5.1 mV and −45.7 ± 7.6 mV, respectively), with curves showing single and narrow peaks. Similarly, a recent study showed that a pH value of 5 was optimal to produce gold nanoparticles that are highly monodisperse and spherical in shape, with a detrimental effect on the size polydispersity at lower pH values [30]. By contrast, the highest pZ dispersion was observed at pH 4.7 (−42.2 ± 35.1 mV), showing a flat distribution curve with three minor peaks that accounts for different populations of citrate-capped AuNPs having a wide range of surface charges. Importantly, the right tail of this curve mostly falls into the region of positive surface charge values (minor peak at 16.87 ± 7.1 mV), revealing a loss on the citrate coverage of the gold surface. These results suggest that one can expect a lesser tendency of partially-protonated citrate ionic species to coordinate with the AuNPs at pH 4.7.

In order to understand the effect of pH on the synthesis reaction, the role of citrate (and protons) must be considered both in terms of the redox reaction forming AuNPs (citrate as oxidizer), as well as from the point of view of its protectant role (citrate as stabilizer). First, looking at the standard reaction mechanism, the initial step of this multiple-step process is the oxidation of citrate yielding dicarboxy acetone (Equation (1)). The second step consist of reduction of auric salt (Au^3+^) to aurous salt (Au^1+^) by accepting the electrons from the citrate oxidation reaction (Equation (2)), and the final step is the disproportionation of aurous species to gold atoms (Au^0^) (Equation (3)):

(1) (O−COCH2)2C(OH)COO−→(O−COCH2)2C=O+CO2+H++2e−

(2) $${\mathrm{AuCl}}_{3}+2e^{-}\to \mathrm{AuCl}+2{\mathrm{Cl}}^{-}$$

(3) $$3\mathrm{AuCl}\to 2\mathrm{Au}+{\mathrm{AuCl}}_{3}$$

The formation of an intermediate pentacoordinate complex of Au^3+^ species with dicarboxyacetone has been proposed, which subsequently decarboxylates to give Au^+^ species [29]. However, the overall stoichiometry of the reduction reaction can be represented as:

(4) 2AuCl3+3(O−COCH2)2C(OH)COO−→2Au0+3(O−COCH2)2C=O+6Cl−+3H++3CO2.

In accordance with Le Chatelier’s principle, as the concentration of protons increases in the solution, the tendency of citrate to oxidize decreases, as well as the availability of electrons to reduce the gold(III) chloride and to form AuNPs. In addition, coordination bonding between the gold surface and adsorbed molecules of citrate consist of carboxyl oxygens that contribute the electron pairs forming covalent bonds [29]. The complexity of the structural arrangement of the citrate layers on AuNPs has been analyzed in several recent studies, showing that citrate could coordinate to the gold through several different binding modes (geometries) that are mostly dictated by one or two of the terminus carboxyl groups [31,32,33]. However, to promote the intermolecular interactions it is required for citrate to diffuse on the gold surface in a preferential fully deprotonated form, since protonated carboxylate groups does not readily adsorb on gold due to electrochemical impediments [33]. Accordingly, the protonation of citrate terminus sites is considered to discourage citrate binding to the gold surface [32], in agreement with our observation of the detrimental effect of pH fluctuations on coverage, stabilization, and production of the AuNPs. However, future work is needed in order to gain a more detailed understanding of these specific sources of variability, by following a more comprehensive and statistically robust approach [34].

## 4. Conclusions

We have demonstrated that under standard experimental conditions of the citrate reduction method, gold nanoparticles are produced in variable concentrations, showing a pH-dependent linear correlation in the range monitored between pH 4.7 and 5.3 (typical pH fluctuation). The AuNP synthesis performance reduced by 46% from 2.4 nM (pH 5.3) to 1.29 nM (pH 4.7), without alteration of size and morphology. The physical characterization of the nanoparticles suggests that slight fluctuations of pH during synthesis can have an adverse effect on citrate oxidation, as well as on the availability of negative charges from carboxylate groups required for an optimal coverage, stabilization, and production of the nanoparticles. Overall, these results provide insight into the effect of the pH over the AuNP synthesis performance of the method, which would be of particular interest for further studies optimizing the reaction conditions in large-scale manufacturing processes.

## Figures and Tables

**Table 1:** Zeta potential, concentration and diameter of AuNPs at pH 4.7, 5.0, and 5.3.

pH	Zeta Potential Mean (mV)	Peak 1 (mV)	Peak 2 (mV)	Peak 3 (mV)	Concentration (nM)	Diameter (nm)
4.7	−42.2 ± 35.1	−58.5 ± 15.2	−26.7 ± 8.2	16.8 ± 7.1	1.29 ± 0.09	13.92 ± 1.45
5.0	−44.9 ± 5.1	−44.9 ± 5.1			1.88 ± 0.03	14.94 ± 1.53
5.3	−45.7 ± 7.6	−45.7 ± 7.6			2.40 ± 0.12	15.50 ± 1.51
